# Supplementary material for: Causal Relationship between Aging and Anorexia Nervosa: A White-Matter-Microstructure-Mediated Mendelian Randomization Analysis
Source: Biomedicines. 2024 Aug 16;12(8):1874. doi: 10.3390/biomedicines12081874 (PMC11351342; doi:10.3390/biomedicines12081874)
Supplement: Supplementary file 1 [file biomedicines-12-01874-s001.zip › Supplementary Figure/Figure S4/Singe SNP plot of MR analysis for TL to ubm-b-1611.pdf]

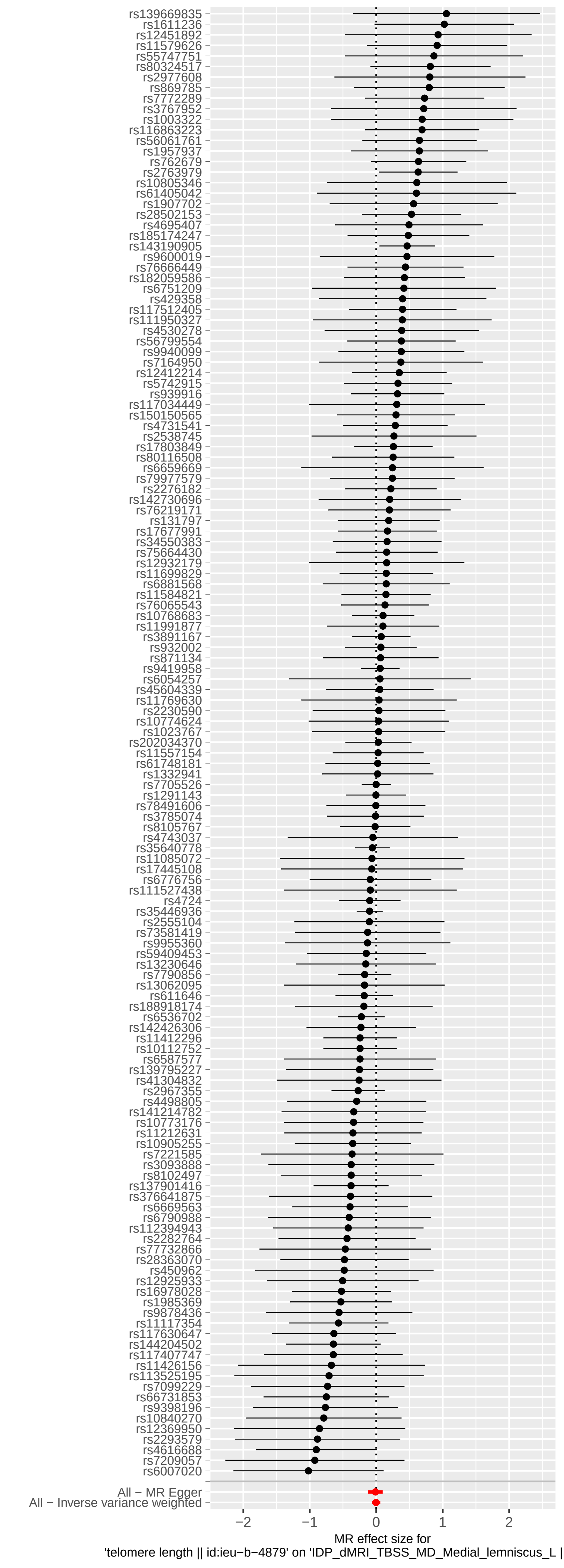

rs139669835
rs1611236
rs12451892
rs11579626
rs55747751
rs80324517
rs2977608
rs869785
rs7772289
rs3767952
rs1003322
rs116863223
rs56061761
rs1957937
rs762679
rs2763979
rs10805346
rs61405042
rs1907702
rs28502153
rs4695407
rs185174247
rs143190905
rs9600019
rs76666449
rs182059586
rs6751209
rs429358
rs117512405
rs111950327
rs4530278
rs56799554
rs9940099
rs7164950
rs12412214
rs5742915
rs939916
rs117034449
rs150150565
rs4731541
rs2538745
rs17803849
rs80116508
rs6659669
rs79977579
rs2276182
rs142730696
rs76219171
rs131797
rs17677991
rs34550383
rs75664430
rs12932179
rs11699829
rs6881568
rs11584821
rs76065543
rs10768683
rs11991877
rs3891167
rs932002
rs871134
rs9419958
rs6054257
rs45604339
rs11769630
rs2230590
rs10774624
rs1023767
rs202034370
rs11557154
rs61748181
rs1332941
rs7705526
rs1291143
rs78491606
rs3785074
rs8105767
rs4743037
rs35640778
rs11085072
rs17445108
rs6776756
rs111527438
rs4724
rs35446936
rs2555104
rs73581419
rs9955360
rs59409453
rs13230646
rs7790856
rs13062095
rs611646
rs188918174
rs6536702
rs142426306
rs11412296
rs10112752
rs6587577
rs139795227
rs41304832
rs2967355
rs4498805
rs141214782
rs10773176
rs11212631
rs10905255
rs7221585
rs3093888
rs8102497
rs137901416
rs376641875
rs6669563
rs6790988
rs112394943
rs2282764
rs77732866
rs28363070
rs450962
rs12925933
rs16978028
rs1985369
rs9878436
rs11117354
rs117630647
rs144204502
rs117407747
rs11426156
rs113525195
rs7099229
rs66731853
rs9398196
rs10840270
rs12369950
rs2293579
rs4616688
rs7209057
rs6007020
All − MR Egger
All − Inverse variance weighted
−2
−1
0
1
2
MR effect size for
'telomere length || id:ieu−b−4879' on 'IDP_dMRI_TBSS_MD_Medial_lemniscus_L |
